# Food security among SNAP participants 2019 to 2021: a cross-sectional analysis of current population survey food security supplement data

**DOI:** 10.1017/jns.2023.32

**Published:** 2023-04-11

**Authors:** Patrick J. Brady, Lisa Harnack, Rachel Widome, Kaitlyn M. Berry, Sruthi Valluri

**Affiliations:** 1 Division of Epidemiology and Community Health, University of Minnesota School of Public Health, Minneapolis, MN, USA; 2 Division of Epidemiology and Community Health, University of Minnesota Twin Cities, Minneapolis, MN, USA; 3 Division of Epidemiology and Community Health, University of Minnesota, Minneapolis, MN, USA; 4 Department of Epidemiology and Community Health, University of Minnesota Medical School, Minneapolis, MN, USA

**Keywords:** Current Population Survey Food Security Supplement, Food insecurity, Food security, Supplemental Nutrition Assistance Program, SNAP

## Abstract

Surveillance data indicate that food security rates increased among Supplemental Nutrition Assistance Program (SNAP) participants during the COVID-19 pandemic (2020 and 2021) compared with pre-pandemic (2019), but this could have been due to increased participation from better resourced households. Our objective was to examine if demographic differences between SNAP-participating households in each year were responsible for the increased prevalence of food secure households. We calculated the observed 30-d food security prevalence among SNAP-participating households for each year. We used indirect standardisation to produce expected 2020 and 2021 prevalences with 2019 as the standard population using household size, income, age, sex, race, Hispanic ethnicity, presence of children, single parent household, metropolitan status and census region. We calculated standardised prevalence ratios (SPRs) to understand if the observed prevalence was higher than expected given any changes in the demographic profile compared to 2019. The Current Population Survey data were collected by the United States Census Bureau and Department of Agriculture. Our sample included 5,245 SNAP-participating households. The observed prevalence of food secure households increased by 3⋅6 percentage points comparing 2019 to 2020 (SPR = 1⋅06, 95 % confidence interval = 1⋅00, 1⋅11) and by 8⋅6 percentage comparing 2019 to 2021 (SPR = 1⋅13, 95 % confidence interval = 1⋅07, 1⋅18). The greater prevalence of food secure SNAP households during the pandemic did not appear to be attributable to socio-demographic differences compared to pre-pandemic. Despite hesitance among policymakers to expand or enhance social safety net programmes, permanently incorporating COVID-19-related policy interventions could lessen food insecurity in years to come.

## Introduction

When the COVID-19 pandemic began in 2020, public health experts were concerned that food insecurity would substantially increase among already vulnerable populations due to rising unemployment and financial instability. Early reports supported this concern^(1–3)^. Nonetheless, 12-month food insecurity rates among all households were stable at 10⋅6 % in both 2019 and 2020 and reduced to 10⋅2 % in 2021 according to the United States Department of Agriculture (USDA) analyses^(4–6)^. The U.S. federal government's policy response to COVID-19, including a variety of economic interventions and enhancing food assistance programmes such as the Supplemental Nutrition Assistance Program (SNAP), may have prevented food insecurity from increasing despite the harsh economic context.

Major changes to SNAP, the largest federal food assistance programme in the United States, included the provision of the maximum benefit level to most households through emergency allotments and a temporary 15 % increase in benefits. Households received an average benefit of approximately $155 per person per month in 2020, which was 19 % higher than 2019^(7)^. While SNAP income eligibility requirements were not altered, policymakers increased access to SNAP by implementing waivers for work requirements for Able-Bodied Adults Without Dependents (ABAWDs), creating exceptions for some previously ineligible college students, and making administrative changes to reduce participation barriers (e.g. no in-person interview required). Administrative data shows that participation in SNAP increased by 12 % from 2019 to 2020^(7)^. Increased accessibility to SNAP in a period of severe economic disruption may have shifted the overall demographics of SNAP participants to include more households with less baseline vulnerability to food insecurity compared to previous years.

In SNAP's long history (see Nestle, 2019 for a summary of the pre-pandemic trajectory of SNAP)^(8)^, the pandemic-related actions in SNAP policy represent significant changes to the programme. Policymakers had not substantially increased SNAP benefits since the 1970s beyond adjustments for inflation or making temporary modifications during economic emergencies (e.g. following the 2007 financial crisis). The combination of emergency allotments and the temporary increase in benefits was considerable. SNAP policy regarding eligibility and access are dictated in large part by state-level politics. As a result, there is a large amount of variability between states in terms of eligibility and administrative policy, including work requirements for ABAWDs, exclusions of vehicles and other asset tests from eligibility determinations and policies regarding categorical eligibility. This leads to differential programme uptake between states^(9,10)^. The pandemic-related changes that broadened eligibility and increased access were widely adopted and may have somewhat equalised the differential uptake between states.

Legislators also implemented multiple policies outside of SNAP to support food and economic security. Policymakers extended unemployment benefits to previously ineligible groups, provided enhanced unemployment benefits of $600 and later $300 per week and implemented a variety of financial supports, including stimulus checks, an eviction moratorium, student loan deferment and enhanced healthcare subsidies. This infusion of resources and relief from regular expenses may have freed up resources that households could direct to food. Non-SNAP policy interventions to the food safety net included providing funds to replace school meals through pandemic-EBT and granting $850 million for The Emergency Food Assistance Program, which provides support for food banks. Details on the numerous policy responses to the pandemic can be found elsewhere^(11,12)^.

Encouragingly, there were declines in the prevalence of food insecure SNAP households in 2020 and 2021. Among households that participated in SNAP for at least one month with incomes less than 130 % of the Federal Poverty Level, the 12-month food insecurity rate was 45⋅4 % in 2020^(5)^ and 39⋅9 % in 2021^(6)^ compared with 49⋅7 % in 2019^(4)^. However, comparing prevalence estimates from annual reports does not account for selection into SNAP. The constellation of food and economic policy interventions during 2020 and 2021, including substantial changes to SNAP benefits and also important but less considerable changes to eligibility and administrative policies, could have given sufficient support to lift many low-income households out of food insecurity despite the wider economic context of the pandemic. Alternatively, socio-demographic differences among income-eligible households participating in SNAP between years may be driving the observed changes in food security prevalence.

Using Current Population Survey (CPS) data, this exploratory study evaluated whether different demographic profiles of SNAP participants in 2020 and 2021 compared to 2019 led to improvements in food security in the SNAP-participating population. We do this by quantifying food security rates using standardisation to make each year comparable on key socio-demographic factors. Examining the extent to which demographics were responsible for observed improvements will help policymakers and anti-hunger advocates better understand observed changes in food security rates in the context of the COVID-19 pandemic.

## Methods

### Data

We used cross-sectional data from the CPS Food Security Supplement (FSS)^(13)^. The CPS is a nationally representative, monthly survey of the non-institutional population aged 15 years and older implemented by the United States Census Bureau and Bureau of Labor Statistics that uses a probability-based, multi-stage, stratified sampling design^(13)^. Households participate in multiple surveys over the course of 16 months, with surveys occurring in the first 4 and final 4 months after being sampled. When a household is selected, a single, knowledgeable member of the household (the household respondent) is interviewed about themselves and all other household members. The CPS uses both in-person and telephone interviews, where in-person interviews generally take place for the first and fifth surveys. A sub-sample of households participate in the FSS in December of each year. The FSS asks questions related to food needs and use of food assistance and includes the 18-item USDA Household Food Security Survey Module (HFSSM)^(14)^. The interview mode does not significantly impact responses to the HFSSM^(15)^. We accessed data through IPUMS^(16)^, an online data repository run by the Minnesota Population Center at the University of Minnesota that provides integrated and harmonised CPS data from 1962 to present^(16)^.

### Study sample

Our sample consists of households that were SNAP participants in November or December of each year. We selected these months to ensure that households were participating in SNAP during the 30-day reference period of our outcome measure. Only households who were completing the FSS for the first time were included (i.e. we included those who completed the supplement in the first 4-month survey cycle and those who completed the supplement in the second 4-month survey cycle but did not complete it in the previous year). We did this to ensure that households would not be included in multiple years and that each year represented an independent cross-section of SNAP-participating households. We also excluded cases with missing data for the HFSSM (*n* 13). This resulted in a total of 5245 households (*n*_2019_ = 2213, *n*_2020_ = 1523, *n*_2021_ = 1509). We also stratify our sample by characteristics that would be associated with receiving non-SNAP-related financial and food support, specifically employment status, receipt of emergency food, and participation in Free or Reduced (FRL) lunch.

### Measures

All households had complete data for each of the measures described in this section. The outcome of the present study was a dichotomous measure of food security in the previous 30 days. This measure is derived from the 18-item HFSSM^(14)^. Households are classified as food secure if responding affirmatively to two or fewer of the items indicating inadequate food access in the prior 30 days. We used the number of months participating in SNAP between March and December in each year as a continuous measure to examine the difference in time enrolled in SNAP in each year. The time period (March to December) for this measure was selected to reflect when COVID-19-related policy changes began to be implemented in 2020. We used the average monthly SNAP benefit per household member as a continuous measure to examine the extent of difference in benefits received each year. This variable was based on a categorical variable indicating a range of benefits received per month for the households with $9 increments. The continuous measure was created by assigning the approximate midpoint value within each category (e.g. the $25–$34 category is assigned a value of $30) and dividing by the household size.

We used multiple measures of respondent- and household-level demographic characteristics. Respondent-level demographics are based on the household respondent. We view the racial self-classification and Hispanic ethnicity measures described below as indicators of socially constructed identities rather than innate characteristics. We use these variables to characterise the SNAP population in terms of the proportion of the respondents who classify themselves as having these identities. Respondent-level demographic characteristics include age (34 and under, 35–59, and 60 and older), sex (male or female), racial self-classification (white, Black, Asian/American Indian/Alaskan Native/Hawaiian/Pacific Islander, multiracial) and Hispanic ethnicity (Hispanic or non-Hispanic). Household-level demographics include household size (continuous), yearly household income (under $20 000, $20 000–$29 999, $30 000–$39 999, $40 000–$49 999, $50 000 and over), whether a child is present in the house (yes or no), whether the household is a single parent household (yes or no), metropolitan status (not identifiable, not in metro area, in metro area) and census region (Northeast, South, Midwest, South).

We also stratified our sample based on three demographic factors: employment status, receipt of emergency food and participation in FRL. Employment status was defined as employed, unemployed (actively looking for work) or not in labour force (neither employed nor unemployed, e.g. retired individuals, students, not seeking employment). For household respondents who were identified as unemployed, we examined the number of weeks unemployed as a continuous variable. Receipt of emergency food from a church, food pantry, food bank or soup kitchen in the previous 30 days and participation in FRL in the previous 30 days were defined as yes/no dichotomous variables.

### Statistical analysis

To assess if there were differences in the demographic characteristics of households participating in SNAP between 2019, 2020 and 2021, we calculated descriptive statistics for each measure and compared years. Next, we examined the observed difference in the prevalence of food secure households in each year. Then, we stratified our sample by employment status, receipt of emergency food and participation in FRL and repeated the descriptive analysis. Finally, we used indirect standardisation^(17)^ on the overall and stratified results to generate the expected prevalence of food secure households in 2020 and 2021 based on 2019 as a reference population using age, sex, racial self-classification, Hispanic ethnicity, household size, income, presence of children, single parent household, metropolitan status and census region. These variables were selected because they have previously been shown to be associated with experiencing food insecurity^(4,5)^. This allowed us to examine whether the observed changes in 2020 and 2021 would be expected given any demographic shifts in the SNAP population between years. We then calculated a standardised prevalence ratio to compare the observed and expected prevalences. A standardised prevalence ratio greater than one represents a larger than expected increase. We calculated standard errors and 95 % confidence intervals for the expected prevalences and standardised prevalence ratios (SPRs) using bootstrapped results under the normal approximation^(17)^. The FSS provides weights at the household level and all analyses were weighted to account for survey design and non-response. All results presented below are weighted means or prevalences. Stata Version 17 was used for all analyses^(18)^.

## Results

### Demographic characteristics

Demographic characteristics are shown in Table 1. In each year, the samples were mostly 35–59 years old, female, white, non-Hispanic, earned under $20 000 per year and had approximately 3 members per household. The proportion of household respondents identifying as Hispanic was higher in 2020 (21⋅7 %) and 2021 (23⋅3 %) compared with 2019 (18⋅1 %). There were fewer households earning $20 000 or less in 2020 (54⋅1 %) and 2021 (56⋅4 %) *v*. 2019 (60⋅5 %). In 2020, more households were earning $50 000 or more (10⋅6 %) compared with 2019 (8⋅2 %), but in 2021, only 8⋅0 % of households earned more than $50 000. The percent of unemployed household respondents nearly doubled from 2019 to 2020 but returned to 2019 levels in 2021. For unemployed household respondents, the number of weeks unemployed was approximately 3 weeks higher in 2020 than 2019 and 6 weeks higher in 2021 than 2019. Receipt of emergency food was higher in 2020 compared to 2019 but returned to 2019 levels in 2021. Participation in FRL showed the opposite trend to receipt of emergency food, with lower participation in 2020 compared to 2019 and returns to a higher level reflective of 2019 in 2021.

**Table 1.** Demographic characteristics of households who participated in SNAP in November or December in 2019 to 2021 presented as % [95 % confidence interval] or mean ± standard deviation based on Current Population Survey Food Security Supplement data

Characteristic	2019 (*n* 2213)	2020 (*n* 1523)	2021 (*n* 1509)
Age			
34 and under	26⋅6 [24⋅2, 29⋅0]	25⋅7 [23⋅1, 28⋅5]	24⋅3 [21⋅7, 27⋅0]
35 to 59	41⋅6 [39⋅2, 44⋅1]	46⋅0 [43⋅0, 48⋅9]	44⋅3 [41⋅4, 47⋅3]
60 and older	31⋅8 [29⋅7, 34⋅1]	28⋅3 [25⋅8, 31⋅0]	31⋅4 [28⋅8, 34⋅2]
Sex			
Male	32⋅8 [30⋅5, 35⋅2]	30⋅0 [27⋅4, 32⋅7]	33⋅9 [31⋅2, 36⋅8]
Female	67⋅2 [64⋅8, 69⋅5]	70⋅0 [67⋅3, 72⋅6]	66⋅1 [63⋅2, 68⋅8]
Racial self-classification			
White	60⋅4 [57⋅8, 62⋅9]	61⋅6 [58⋅6, 64⋅6]	61⋅4 [58⋅4, 64⋅3]
Black	31⋅1 [28⋅7, 33⋅7]	29⋅7 [26⋅9, 32⋅6]	30⋅6 [27⋅8, 33⋅6]
Asian, American Indian/Alaskan Native, Hawaiian/Pacific Islander	5⋅5 [4⋅5, 6⋅7]	4⋅7 [3⋅6, 6⋅1]	5⋅6 [4⋅4, 7⋅0]
Multiracial	3⋅0 [2⋅2, 4⋅0]	4⋅0 [2⋅9, 5⋅5]	2⋅4 [1⋅7, 3⋅6]
Hispanic ethnicity			
Yes	18⋅1 [16⋅2, 20⋅1]	21⋅9 [19⋅4, 24⋅5]	23⋅3 [20⋅9, 26⋅0]
No	81⋅9 [79⋅9, 83⋅8]	78⋅1 [75⋅5, 80⋅6]	76⋅7 [74⋅0, 79⋅1]
Employment status			
Employed	31⋅0 [28⋅7, 33⋅4]	31⋅1 [28⋅4, 33⋅9]	34⋅3 [31⋅5, 37⋅2]
Unemployed	5⋅9 [4⋅8, 7⋅3]	11⋅6 [9⋅8, 13⋅7]	6⋅4 [5⋅1, 8⋅0]
Not in labour force	63⋅1 [60⋅6, 65⋅5]	57⋅4 [54⋅4, 60⋅3]	59⋅4 [56⋅4, 62⋅3]
Week unemployed (*n* 375)	22⋅2 ± 20⋅3	25⋅4 ± 17⋅9	29⋅2 ± 28⋅6
Emergency food use in last 30 d			
Yes	32⋅5 [30⋅2, 34⋅9]	39⋅8 [36⋅9, 42⋅7]	28⋅6 [26⋅0, 31⋅3]
No	67⋅5 [65⋅1, 69⋅8]	60⋅2 [57⋅3, 63⋅1]	71⋅4 [68⋅7, 74⋅0]
Free or reduced lunch participant in last 30 d			
Yes	30⋅5 [28⋅2, 32⋅9]	20⋅3 [18⋅1, 22⋅7]	29⋅5 [26⋅9, 32⋅3]
No	69⋅5 [67⋅1, 71⋅8]	79⋅7 [77⋅3, 81⋅9]	70⋅5 [67⋅7, 73⋅1]
Household size	2⋅7 ± 1⋅5	2⋅9 ± 1⋅5	2⋅6 ± 1⋅4
Yearly income			
Under $20 000	60⋅5 [58⋅0, 62⋅9]	53⋅8 [50⋅8, 56⋅7]	56⋅4 [53⋅4, 59⋅3]
$20 000–$29 999	16⋅7 [15⋅0, 18⋅7]	19⋅2 [16⋅9, 21⋅6]	19⋅5 [17⋅2, 22⋅0]
$30 000–$39 999	10⋅5 [9⋅1, 12⋅2]	12⋅2 [10⋅4, 14⋅3]	11⋅3 [9⋅5, 13⋅3]
$40 000–$49 999	4⋅1 [3⋅2, 5⋅2]	4⋅1 [3⋅1, 5⋅4]	4⋅9 [3⋅8, 6⋅3]
$50 000+	8⋅2 [6⋅8, 9⋅8]	10⋅8 [9⋅0, 12⋅8]	8⋅0 [6⋅5, 9⋅9]
Children in household			
Yes	48⋅0 [45⋅5, 50⋅5]	45⋅5 [51⋅5, 57⋅4]	47⋅4 [44⋅4, 50⋅4]
No	52⋅0 [49⋅5, 54⋅5]	45⋅5 [42⋅6, 48⋅5]	52⋅6 [49⋅6, 55⋅6]
Single parent household			
Yes	34⋅3 [31⋅9, 36⋅7]	38⋅8 [35⋅9, 41⋅8]	34⋅0 [31⋅3, 36⋅9]
No	65⋅7 [63⋅3, 68⋅1]	61⋅2 [58⋅2, 64⋅1]	66⋅0 [63⋅1, 68⋅7]
Metropolitan status			
Not identified	1⋅1 [0⋅7, 1⋅6]	1⋅3 [0⋅8, 2⋅0]	0⋅3, 1⋅2]0⋅6
Not in metro area	16⋅7 [15⋅1, 18⋅5]	16⋅7 [14⋅8, 18⋅8]	16⋅0 [14⋅1, 18⋅1]
In metro area	82⋅2 [80⋅4, 83⋅9]	82⋅0 [79⋅9, 84⋅0]	83⋅3 [81⋅2, 85⋅3]
U.S. Census region			
Northeast	19⋅4 [17⋅5, 21⋅5]	17⋅4 [15⋅2, 19⋅9]	19⋅4 [17⋅1, 22⋅2]
Midwest	21⋅3 [19⋅3, 23⋅6]	17⋅9 [15⋅7, 20⋅3]	19⋅7 [17⋅4, 22⋅2]
South	40⋅3 [37⋅8, 42⋅7]	44⋅2 [41⋅3, 47⋅2]	40⋅0 [37⋅1, 43⋅0]
West	19⋅0 [17⋅2, 20⋅9]	20⋅4 [18⋅3, 22⋅8]	20⋅9 [18⋅7, 23⋅2]
Months participating in SNAP March–December	8⋅9 ± 2⋅0	8⋅8 ± 2⋅2	8⋅8 ± 2⋅2
SNAP average monthly benefit per household member	$92⋅46 ± $53⋅25	$109⋅93 ± $53⋅95	$143⋅72 ± $79⋅30

Note 1: Households were included if (1) they participated in SNAP during November or December and (2) they were completing the Current Population Survey Food Security Supplement for the first time.

Note 2: Respondent-level demographics (age, sex, race, ethnicity and employment) were based on the household respondent.

There were no differences in the number of months households were enrolled in SNAP between years, with values of 8⋅9 (sd = 2⋅0), 8⋅8 (sd = 2⋅1) and 8⋅8 (sd = 2⋅2) months. SNAP monthly benefits per household member increased from $92⋅46 (sd = $53⋅25) in 2019 to $110⋅74 (sd = $55⋅68) in 2020 and $143⋅72 (sd = $79⋅30) in 2021. This is approximately a 20 % increase in self-reported SNAP monthly benefits per household member from 2019 to 2020 and an approximately 30 % increase from 2020 to 2021.

### 30-day food security status

The overall prevalences of food secure SNAP-participating households in each year are shown in Table 2. The observed prevalence of food security in SNAP-participating households in 2019, 2020 and 2021 were 70⋅0, 73⋅6 and 78⋅6 %, respectively – a 3⋅6 percentage point increase between 2019 and 2020 and a further 5⋅0 percentage point increase from 2020 to 2021. The expected prevalences of food security in 2020 and 2021 based on standardisation were 69⋅7 and 69⋅8 %. The standardised prevalence ratio for food secure households was 1⋅06 (95 % CI 1⋅00, 1⋅11) in 2020 and 1⋅13 (95 % CI 1⋅07, 1⋅18), indicating that the observed prevalences of food security in 2020 and 2021 were greater than the prevalence expected based on socio-demographic factors included in the model.

**Table 2.** Food security status in the previous 30 d among households who participated in SNAP in November or December in 2019 and 2020 presented as % [95 % confidence interval] and standardised prevalence ratios [95 % confidence interval] from Current Population Survey Food Security Supplement data

30-d food security	2019	2020	2021
SNAP-participating households	*n* 2213	*n* 1523	*n* 1509
Food secure (observed)	70⋅0 [67⋅6, 72⋅3]	73⋅6 [70⋅8, 76⋅1]	78⋅6 [76⋅0, 81⋅0]
Food secure (expected)		69⋅7 [67⋅3, 72⋅0]	69⋅8 [67⋅5, 72⋅2]
Standardised prevalence ratio		1⋅06 [1⋅00, 1⋅11]	1⋅13 [1⋅07, 1⋅18]

Note 1: Expected values were generated using indirect standardisation to 2019 based on household size, yearly income, age, sex, racial self-classification, Hispanic ethnicity, presence of children, single parent household, metropolitan status and census region; 95 % confidence intervals for the expected prevalence and standardised prevalence ratio were generated using bootstrapped results.

Note 2: Households were included if (1) they participated in SNAP during November or December of either year and (2) they were completing the Current Population Survey Food Security Supplement for the first time.

### 30-day food security status stratified by employment status

The prevalence of food secure households in each year stratified by employment status are shown in Table 3. In 2020, there was a 5⋅7 percentage points higher prevalence of food secure households in comparison to 2019 for households where the respondent was not in the labour force. In 2021, there was an 8⋅7 percentage points higher prevalence of food secure households in comparison to 2019 for households where the respondent was not in the labour force. There were smaller increases in 2020 for households where the household respondent was employed (+1⋅4 percentage points) or unemployed (+2⋅2 percentage points), but much larger increases in 2021 (+5⋅8 percentage points for employed households and +17⋅4 percentage points for unemployed households). For 2020, the SPRs for food secure households were 1⋅06 (95 % CI 0⋅97, 1⋅14) for households where the household respondent was employed, 1⋅14 (95 % CI 0⋅81, 1⋅47) for households where the household respondent was unemployed and 1⋅08 (95 % CI 1⋅01, 1⋅15) for households where the household respondent was not in the labour force. For 2021, the SPRs for food secure households were 1⋅11 (95 % CI 1⋅02, 1⋅19) for households where the household respondent was employed, 1⋅52 (95 % CI 1⋅02, 2⋅02) for households where the household respondent was unemployed and 1⋅08 (95 % CI 1⋅05, 1⋅19) for households where the household respondent was not in the labour force.

**Table 3.** Food security status in the previous 30 d among households who participated in SNAP in November or December in 2019 and 2020 presented as % [95 % confidence interval] and standardised prevalence ratios [95 % confidence interval] from Current Population Survey Food Security Supplement data stratified by employment status

30-d food security	2019	2020	2021
Employed	*n* 641	*n* 456	*n* 488
Food secure (observed)	77⋅9 [73⋅8, 81⋅6]	79⋅7 [74⋅9, 83⋅7]	83⋅7 [79⋅3, 87⋅2]
Food secure (expected)		75⋅3 [70⋅9, 79⋅8]	75⋅5 [71⋅2, 79⋅9]
Standardised prevalence ratio		1⋅06 [0⋅97, 1⋅14]	1⋅11 [1⋅02, 1⋅19]
Unemployed	*n* 124	*n* 156	*n* 95
Food secure (observed)	61⋅5 [50⋅4, 71⋅6]	63⋅1 [54⋅1, 71⋅2]	78⋅9 [68⋅0, 86⋅8]
Food secure (expected)		55⋅4 [42⋅4, 68⋅4]	51⋅9 [36⋅8. 67⋅0]
Standardised prevalence ratio		1⋅14 [0⋅81, 1⋅47]	1⋅52 [1⋅02, 2⋅02]
Not in labour force	*n* 1448	*n* 911	*n* 926
Food secure (observed)	66⋅9 [63⋅9, 69⋅8]	72⋅4 [68⋅7, 75⋅7]	75⋅6 [72⋅1, 78⋅8]
Food secure (expected)		67⋅0 [64⋅1, 69⋅9]	67⋅5 [64⋅5, 70⋅4]
Standardised prevalence ratio		1⋅08 [1⋅01, 1⋅15]	1⋅12 [1⋅05, 1⋅19]

Note 1: Expected values were generated using indirect standardisation to 2019 based on household size, yearly income, age, sex, racial self-classification, Hispanic ethnicity, presence of children, single parent household, metropolitan status and census region; 95 % confidence intervals for the expected prevalence and standardised prevalence ratio were generated using bootstrapped results.

Note 2: Households were included if (1) they participated in SNAP during November or December of either year and (2) they were completing the Current Population Survey Food Security Supplement for the first time.

### 30-day food security status stratified by receipt of emergency food and FRL participation

The prevalence of food secure households in each year stratified by receipt of emergency food and participation in FRL are shown in Table 4. For households that received emergency food, the prevalence of food security was 7⋅0 percentage points higher in 2020 and 10⋅4 percentage points higher in 2021 in comparison to 2019. For households that did not receive emergency food, the prevalence of food secure households was 4⋅1 percentage points higher in 2020 and 6⋅6 percentage points higher in 2021 compared to 2019. The standardised prevalence ratio for food secure households was 1⋅12 (95 % CI 1⋅00, 1⋅25) for 2020 and 1⋅18 (95 % CI 1⋅04, 1⋅32) for households that received emergency food. The SPRs for households that did not receive emergency food were 1⋅06 (95 % CI 1⋅00, 1⋅11) for 2020 and 1⋅09 (95 % CI 1⋅04, 1⋅14) for 2021. Compared to 2019, the prevalence of food secure households was 7⋅4 and 9⋅0 percentage points higher in 2020 and 2021 for FRL participants. For non-participants, the prevalence of food secure households was 3⋅1 percentage points higher in 2020 and 8⋅5 percentage points higher in 2021 compared to 2019. The standardised prevalence ratio for food secure households was 1⋅13 (95 % CI 1⋅02, 1⋅23) for 2020 and 1⋅14 (95 % CI 1⋅05, 1⋅24) for 2021 for households that participated in FRL and 1⋅04 (95 % CI 0⋅97, 1⋅10) for 2020 and 1⋅12 (95 % CI 1⋅05, 1⋅18) for 2021 for households that did not participate in FRL.

**Table 4.** Food security status in the previous 30 d among households who participated in SNAP in November or December in 2019 and 2020 presented as % [95 % confidence interval] and standardised prevalence ratios [95 % confidence interval] from Current Population Survey Food Security Supplement data stratified by receipt of emergency food and participation in Free or Reduced Lunch (FRL)

30-d food security	2019	2020	2021
Received emergency food	*n* 739	*n* 594	*n* 441
Food secure (observed)	54⋅4 [50⋅0, 58⋅7]	61⋅4 [56⋅6, 65⋅9]	64⋅8 [59⋅3, 69⋅9]
Food secure (expected)		54⋅6 [49⋅9, 59⋅4]	54⋅9 [50⋅3, 59⋅6]
Standardised prevalence ratio		1⋅12 [1⋅00, 1⋅25]	1⋅18 [1⋅04, 1⋅32]
Did not receive emergency food	*n* 1474	*n* 929	*n* 1068
Food secure (observed)	77⋅5 [74⋅8, 80⋅0]	81⋅6 [78⋅4, 84⋅5]	84⋅1 [81⋅3, 86⋅6]
Food secure (expected)		77⋅3 [74⋅7, 79⋅9]	77⋅3 [74⋅7, 79⋅9]
Standardised prevalence ratio		1⋅06 [1⋅00, 1⋅11]	1⋅09 [1⋅04, 1⋅14]
Free and Reduced Lunch participant	*n* 641	*n* 329	*n* 437
Food secure (observed)	72⋅7 [68⋅3, 76⋅7]	80⋅1 [74⋅5, 84⋅7]	81⋅7 [76⋅9, 85⋅6]
Food secure (expected)		71⋅0 [66⋅3, 75⋅6]	71⋅4 [67⋅0, 75⋅8]
Standardised prevalence ratio		1⋅13 [1⋅02, 1⋅23]	1⋅14 [1⋅05, 1⋅24]
Free and Reduced Lunch non-participant	*n* 1572	*n* 1194	*n* 1072
Food secure (observed)	68⋅8 [66⋅0, 71⋅5]	71⋅9 [68⋅7, 74⋅8]	77⋅3 [74⋅1, 80⋅2]
Food secure (expected)		69⋅4 [66⋅4, 72⋅5]	69⋅2 [66⋅3, 72⋅0]
Standardised prevalence ratio		1⋅04 [0⋅97, 1⋅10]	1⋅12 [1⋅05, 1⋅18]

Note 1: Expected values were generated using indirect standardisation to 2019 based on household size, yearly income, age, sex, racial self-classification, and Hispanic ethnicity, presence of children, single parent household, metropolitan status and census region; 95 % confidence intervals for the expected prevalence and standardised prevalence ratio were generated using bootstrapped results.

Note 2: Households were included if (1) they participated in SNAP during November or December of either year and (2) they were completing the Current Population Survey Food Security Supplement for the first time.

## Discussion

Our results indicate food security among SNAP-participating households was higher in 2020 and 2021 compared to 2019, and this difference did not appear to be attributable to changes in the socio-demographics of who was participating in SNAP. Furthermore, we saw larger increases in the prevalence of food secure households in 2021, with consistent trends across employment status, receipt of emergency food and participation in FRL. While improvements in food security status from 2019 to 2021 among SNAP participants have been documented elsewhere^(4–6)^, it was unclear if this was due to changes in the demographic profile of SNAP participants caused by extended eligibility, reduced barriers to participation, or increased enrolment in safety net programmes including SNAP driven by the pandemic^(7,19)^. Our results indicate that changes in demographic characteristics were not driving the improvements in food security status seen among SNAP-participating households in 2020 and 2021.

Because the COVID-19 pandemic severely disrupted employment^(20)^, policymakers expanded eligibility for unemployment assistance (e.g. to part-time workers and the self-employed) and provided enhanced unemployment benefits. Previous research has shown that the enhanced unemployment benefits improved food security and reduced economic concerns among recipients^(21,22)^. In the present study, we were not able to determine whether unemployed adults in the household received unemployment benefits. In 2020, we observed greater than expected increases in the prevalence of food secure households only among households not in the labour force. This may be partially due to the smaller sample sizes in the employed and unemployed strata. Alternatively, for respondents unemployed during the pandemic, unemployment insurance and other policy interventions may not fully counteract an acute loss of wages. In contrast, respondents who are not in the workforce may be less likely to have sudden changes to their incomes; thus, additional SNAP benefits may have induced greater than expected improvements in food security. In 2021, we saw greater than expected increases in the prevalence of food secure SNAP-participating households across strata based on employment status, perhaps due to wider economic trends in 2021, which are also reflected in the decreased nationwide food insecurity rate in 2021^(6)^. Further research is needed to examine the extent unemployment benefits supported the food security of SNAP participants in the pandemic.

Increases in the prevalence of food secure households for 2020 and 2021 were larger than expected for both households that did and households that did not receive emergency food but were greater in magnitude for those that received emergency food. This may be due to the support provided by emergency food providers but may also be due to households with greater resources using emergency food resources during the pandemic. The SPRs for FRL participants also showed higher than expected improvements in both years, but only greater than expected improvements in 2021 for non-participants. Interpreting these results is complicated by the fact that measurement of FRL participation may have been affected by pandemic-related changes to the programme (e.g. households picking meals up from school may not consider that to be participation in FRL). Similarly, pandemic-EBT substituted for FRL for many households, which may partially explain the reported decline in FRL participation. Despite this, these results suggest that economic policy interventions provided to FRL participants contributed to the food security of these households.

Even with respondents reporting an approximate 20 % increase in monthly SNAP benefits and a wide variety of economic policy interventions delivered in 2020, over a quarter of SNAP-participating households remained food insecure. In 2021, despite the continuation of many of the economic policy interventions and reporting even higher benefits compared to 2020, over one in five of SNAP-participating households remained food insecure. This is consistent with previous studies prior to the pandemic showing SNAP participation reduces but does not eliminate food insecurity^(23–29)^. Future research should aim to understand how an enhanced food safety could more adequately help households meet their nutrition needs.

The present study examined a series of cross-sectional surveys on food security among SNAP participants and as such was not able to determine if the policies changed the trajectories of the risk of experiencing food insecurity for individual households over time. Even with this limitation, using standardisation in this descriptive analysis allowed us to rule out changes in measured demographic factors as an explanation for observed improvements in food security. More rigorous designs should be used to study the specific impacts of policy interventions suggested by the preliminary results presented here. Another limitation of the present study is that we were unable to directly assess the impact of specific policy responses on household food security status, for example, the effect of receiving pandemic-EBT or unemployment benefits. Even with this limitation, by stratifying our results, we were able to compare the prevalence of food secure households among groups that would or would not have been exposed to certain policy interventions. Finally, while our sample is representative of SNAP households, these results should not be generalised to other low-income populations of interest.

## Conclusion

These results show that improvements in food security among SNAP participants were not driven by changing socio-demographics of participants. The variety of pandemic-related policy interventions to prevent food and economic insecurity may have been beneficial to SNAP participants, but further research is needed to establish those relationships. More adequately meeting the basic food needs of low-income households should be a priority among policymakers and, as indicated by the food security rates reported here and consistently shown in other research^(23–29)^, more ambitious initiatives are needed to fully eliminate food insecurity among SNAP participants. Exploring the impact of increasing SNAP benefit levels, increasing accessibility to federal nutrition programmes, supporting the emergency food system and allowing for flexibility in childhood nutrition programmes should be a priority. Continuing to implement and expand improvements to the social safety net while evaluating the extent to which these improvements influence both selection into the programme and programme outcomes has the potential to improve food security, which would be an important public health achievement.
